# *catena*-Poly[[[triaqua­(4,5-diaza­fluorene-9-one)cadmium]-μ-benzene-1,3-dicarboxyl­ato] dihydrate]

**DOI:** 10.1107/S1600536809031237

**Published:** 2009-08-15

**Authors:** Xiao-Ping Li, Wei Fang, Ze-Min Mei, Xiang-Jun Jin, Wen-Liang Qi

**Affiliations:** a Department of Chemistry, Baicheng Normal University, Baicheng 137000, People’s Republic of China; b Siping Academy of Science and Technology, Siping 136000, People’s Republic of China

## Abstract

In the title compound, {[Cd(C_8_H_4_O_4_)(C_11_H_6_N_2_O)(H_2_O)_3_]·2H_2_O}_*n*_, the Cd^II^ atom is seven-coordinated by two N atoms from the phenanthroline-derived 4,5-diaza­fluorene-9-one ligand, two O atoms from one bidentate benzene-1,3-dicarboxyl­ate ligand and three O atoms from the three water mol­ecules in a distorted penta­gonal-bipyramidal arrangement. Moreover, there are two dissociative water mol­ecules in each unit. Neighbouring units inter­act through π–π inter­actions [centroid–centroid distances = 3.325 (3) and 3.358 (4) Å] and O—H⋯O hydrogen-bonding, resulting in a two-dimensional network extending parallel to (001).

## Related literature

The 1,10-phenanthroline (phen) ligand has been widely used to build novel supra­molecular architectures through its aromatic π–π inter­ations, see: Chen & Liu (2002 ▶). The phen derivative 4,5-diaza­fluorene-9-one was recently shown to form a coordination polymer with a distinctive supra­molecular architecture, see: Kraft *et al.* (2002 ▶). For the ligand synthesis, see: Henderson *et al.* (1984 ▶). 
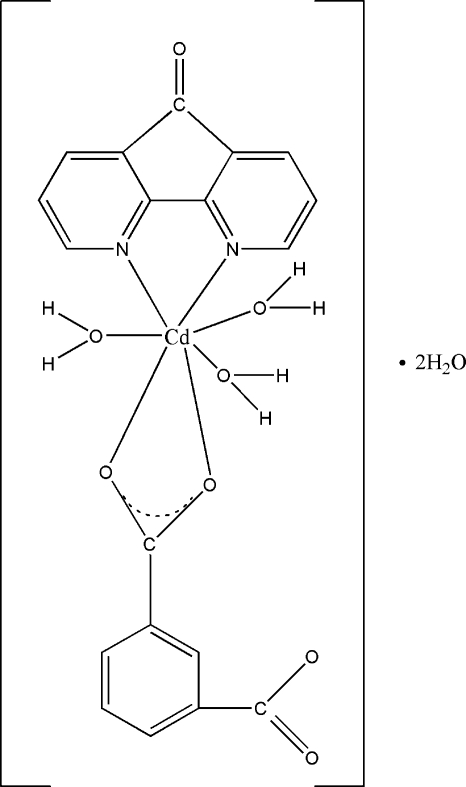


## Experimental

### 

#### Crystal data

[Cd(C_8_H_4_O_4_)(C_11_H_6_N_2_O)(H_2_O)_3_]·2H_2_O
*M* *_r_* = 548.78
Triclinic, 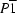

*a* = 6.9383 (10) Å
*b* = 10.8070 (16) Å
*c* = 14.429 (2) Å
α = 96.268 (2)°
β = 92.602 (2)°
γ = 102.019 (2)°
*V* = 1049.3 (3) Å^3^
*Z* = 2
Mo *K*α radiation
μ = 1.10 mm^−1^
*T* = 293 K
0.34 × 0.29 × 0.20 mm

#### Data collection

Bruker APEXII diffractometer
Absorption correction: multi-scan (*SADABS*; Bruker, 1998 ▶) *T*_min_ = 0.697, *T*_max_ = 0.804
5319 measured reflections
3804 independent reflections
3260 reflections with *I* > 2σ(*I*)
*R*_int_ = 0.017

#### Refinement

*R*[*F* ^2^ > 2σ(*F* ^2^)] = 0.037
*wR*(*F* ^2^) = 0.093
*S* = 1.05
3804 reflections
284 parameters
H-atom parameters constrained
Δρ_max_ = 1.39 e Å^−3^
Δρ_min_ = −0.64 e Å^−3^

### 

Data collection: *APEX2* (Bruker, 1998 ▶); cell refinement: *SAINT* (Bruker, 1998 ▶); data reduction: *SAINT*; program(s) used to solve structure: *SHELXS97* (Sheldrick, 2008 ▶); program(s) used to refine structure: *SHELXL97* (Sheldrick, 2008 ▶); molecular graphics: *SHELXL97*; software used to prepare material for publication: *SHELXTL* (Sheldrick, 2008 ▶).

## Supplementary Material

Crystal structure: contains datablocks global, I. DOI: 10.1107/S1600536809031237/jh2092sup1.cif

Structure factors: contains datablocks I. DOI: 10.1107/S1600536809031237/jh2092Isup2.hkl

Additional supplementary materials:  crystallographic information; 3D view; checkCIF report

## Figures and Tables

**Table 1:** Selected geometric parameters (Å, °)

Cd1—O7	2.271 (3)
Cd1—O6	2.326 (3)
Cd1—O2	2.354 (3)
Cd1—O5	2.368 (3)
Cd1—O1	2.441 (3)
Cd1—N2	2.472 (3)
Cd1—N1	2.492 (3)

**Table 2:** Hydrogen-bond geometry (Å, °)

*D*—H⋯*A*	*D*—H	H⋯*A*	*D*⋯*A*	*D*—H⋯*A*
O5—H*O*5*A*⋯O3^i^	0.85	1.95	2.728 (4)	151
O5—H*O*5*B*⋯O4^ii^	0.96	2.06	2.933 (4)	151
O6—H*O*6*A*⋯O*W*1^iii^	0.92	1.86	2.776 (5)	175
O6—H*O*6*B*⋯O4^ii^	0.99	1.70	2.675 (4)	171
O7—H*O*7*A*⋯O4^iv^	0.91	1.96	2.744 (4)	143
O7—H*O*7*B*⋯O*W*2	0.91	1.87	2.757 (4)	162
O*W*1—H*W*1*A*⋯O8^v^	0.89	2.07	2.903 (5)	156
O*W*1—H*W*1*B*⋯O1^vi^	0.90	2.12	2.824 (5)	135
O*W*2—H*W*2*A*⋯O3^vii^	0.99	1.80	2.769 (4)	168
O*W*2—H*W*2*B*⋯O2^viii^	0.95	1.99	2.936 (5)	173

Symmetry codes: (i) 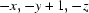; (ii) 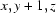; (iii) 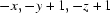; (iv) 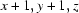; (v) 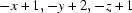; (vi) 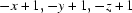; (vii) 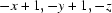; (viii) 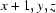.

**Fig. 1.**
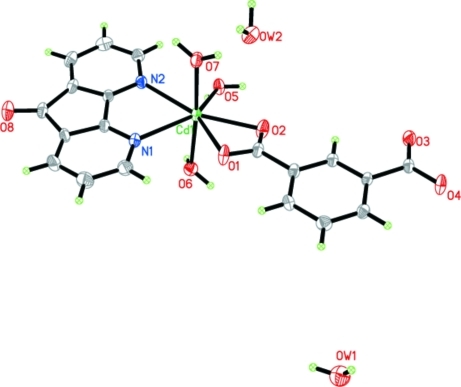
view of the local coordination of Cd(II) with the atomic numbering scheme. Displacement ellipsoids are drawn at the 30% probability level. (arbitrary spheres for the H atoms).

**Fig. 2.**
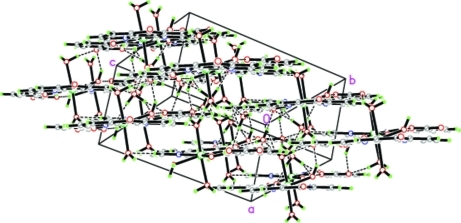
A view of the two-dimensional supramolecular structure of (I) generated by π-π interactions.

## supplementary crystallographic information

### Comment

The 1,10-phenanthroline (phen) ligand has been widely used to build novel supramolecular architectures through its aromatic π-π interations (Chen & Liu, 2002). The phen derivative 4,5-diazafluorene-9-one (C_11_H_6_N_2_O; *L*), was recently shown to form a coordination polymer with a distinctive supramolecular architecture (Kraft *et al.*, 2002). We selected benzene-1,3-dicarboxylate (C_8_H_4_O_4_^2-^; 1,3-BDC) to act as a metal-metal linker in its deprotonated form and *L* as a secondary ligand, generating the title compound, [Cd(C_11_H_6_N_2_O)(C_8_H_4_O_4_)(H_2_O)_3_.2H_2_O], a new coordinationg polymer, which is reported here. In compound (I), the Cd^II^ atom of unit is surrounded by two N atoms derived from the bidentate *L* ligand, two O atom from a bidentate 1,3-BDC ligand and three O atoms from three H_2_O moleculars. This results in a very distorted CdN_2_O_5_ pentagonal bipyramid with the donor atoms of both the bidentate species occupying both an equatorial and an axial site (Table 1, Fig.1). The average Cd—O and Cd—N distances are 2.352 (3) and 2.482 (3) Å, respectively. Neighbouring units in (I) are connectede through π-π interactions between *L* ligands with π-π stacking distances of 3.325 (3) and 3.358 (4) Å, resulting in a two-dimensional supramolecular structure. Finally, interunit OW—H···O hydrogen bonds (Table 2) complete the structure of (I).

### Experimental

Ligand *L* was synthesized according to the literature method. (Henderson *et al.*, 1984). A mixture of CdCl_2_ (0.3 mmol), *L*(0.1 mmol) and H_2_1,3-BDC (0.3 mmol) in distilled water (30 ml) was stirred thoroughly for 1 h at ambient temperature. The pH was adjusted to 7.5 with aqueous NaOH solution. The suspension was then sealed in a Teflon-lined stainless steel reaction vessel (40 ml). The reaction was performed under autogeneous pressure and static conditions in an oven at 443 K for 4.5 d. The vessel was then cooled slowly inside the oven to 298 K at a rate of 5 K h^-1^ before opening: yellow crystals of (I) were collected.

### Refinement

All H atoms on C atoms were generated geometrically and refined as riding atoms with C—H= 0.93Å and *U*_iso_(H)= 1.2 times *U*_eq_(C).

### Crystal data

**Table:**

[Cd(C_8_H_4_O_4_)(C_11_H_6_N_2_O)(H_2_O)_3_]·2H_2_O	*Z* = 2
*M**_r_* = 548.78	*F*(000) = 552.0
Triclinic, *P*1	*D*_x_ = 1.737 Mg m^−^^3^
Hall symbol: -P 1	Mo *K*α radiation, λ = 0.71073 Å
*a* = 6.9383 (10) Å	Cell parameters from 25 reflections
*b* = 10.8070 (16) Å	θ = 7.5–15°
*c* = 14.429 (2) Å	µ = 1.10 mm^−^^1^
α = 96.268 (2)°	*T* = 293 K
β = 92.602 (2)°	Block, yellow
γ = 102.019 (2)°	0.34 × 0.29 × 0.20 mm
*V* = 1049.3 (3) Å^3^	

### Data collection

**Table:**

Bruker APEXII diffractometer	3804 independent reflections
Radiation source: fine-focus sealed tube	3260 reflections with *I* > 2σ(*I*)
graphite	*R*_int_ = 0.017
Detector resolution: 0 pixels mm^-1^	θ_max_ = 25.3°, θ_min_ = 1.4°
ω scans	*h* = −8→6
Absorption correction: multi-scan (*SADABS*; Bruker, 1998)	*k* = −12→12
*T*_min_ = 0.697, *T*_max_ = 0.804	*l* = −17→15
5319 measured reflections	

### Refinement

**Table:**

Refinement on *F*^2^	Primary atom site location: structure-invariant direct methods
Least-squares matrix: full	Secondary atom site location: difference Fourier map
*R*[*F*^2^ > 2σ(*F*^2^)] = 0.037	Hydrogen site location: inferred from neighbouring sites
*wR*(*F*^2^) = 0.093	H-atom parameters constrained
*S* = 1.05	*w* = 1/[σ^2^(*F*_o_^2^) + (0.0466*P*)^2^ + 0.937*P*] where *P* = (*F*_o_^2^ + 2*F*_c_^2^)/3
3804 reflections	(Δ/σ)_max_ < 0.001
284 parameters	Δρ_max_ = 1.39 e Å^−^^3^
0 restraints	Δρ_min_ = −0.64 e Å^−^^3^

### Special details

**Table:**

Geometry. All e.s.d.'s (except the e.s.d. in the dihedral angle between two l.s. planes) are estimated using the full covariance matrix. The cell e.s.d.'s are taken into account individually in the estimation of e.s.d.'s in distances, angles and torsion angles; correlations between e.s.d.'s in cell parameters are only used when they are defined by crystal symmetry. An approximate (isotropic) treatment of cell e.s.d.'s is used for estimating e.s.d.'s involving l.s. planes.
Refinement. Refinement of *F*^2^ against ALL reflections. The weighted *R*-factor *wR* and goodness of fit *S* are based on *F*^2^, conventional *R*-factors *R* are based on *F*, with *F* set to zero for negative *F*^2^. The threshold expression of *F*^2^ > σ(*F*^2^) is used only for calculating *R*-factors(gt) *etc*. and is not relevant to the choice of reflections for refinement. *R*-factors based on *F*^2^ are statistically about twice as large as those based on *F*, and *R*- factors based on ALL data will be even larger.

### Fractional atomic coordinates and isotropic or equivalent isotropic displacement parameters (Å^2^)

**Table:**

	*x*	*y*	*z*	*U*_iso_*/*U*_eq_	
Cd1	0.41915 (4)	0.92090 (2)	0.19901 (2)	0.03260 (12)	
O1	0.4624 (5)	0.7585 (3)	0.2984 (2)	0.0506 (8)	
O2	0.2646 (5)	0.7034 (3)	0.1714 (2)	0.0507 (8)	
O3	0.0030 (5)	0.2413 (3)	0.0709 (2)	0.0466 (7)	
O4	0.0050 (4)	0.1021 (2)	0.1719 (2)	0.0448 (7)	
O5	0.2170 (4)	0.9328 (3)	0.06495 (19)	0.0423 (7)	
HO5A	0.1523	0.8617	0.0376	0.051*	
HO5B	0.1310	0.9904	0.0785	0.051*	
O6	0.1543 (4)	0.9578 (3)	0.28319 (19)	0.0409 (7)	
HO6A	0.0652	0.8878	0.2978	0.049*	
HO6B	0.0857	1.0067	0.2439	0.049*	
O7	0.6983 (4)	0.8944 (3)	0.1303 (2)	0.0423 (7)	
HO7A	0.7909	0.9616	0.1164	0.051*	
HO7B	0.7246	0.8150	0.1185	0.051*	
O8	0.8494 (5)	1.5000 (3)	0.3739 (3)	0.0547 (8)	
OW1	0.1330 (6)	0.2440 (4)	0.6739 (3)	0.0680 (10)*	
HW1A	0.1594	0.3283	0.6755	0.082*	
HW1B	0.2287	0.1998	0.6674	0.082*	
OW2	0.8579 (5)	0.6822 (3)	0.0952 (2)	0.0534 (8)	
HW2A	0.9077	0.6971	0.0335	0.064*	
HW2B	0.9856	0.6831	0.1226	0.064*	
N1	0.6184 (5)	1.0507 (3)	0.3377 (2)	0.0365 (8)	
N2	0.5214 (5)	1.1420 (3)	0.1616 (2)	0.0328 (7)	
C1	0.6800 (7)	1.0281 (4)	0.4218 (3)	0.0478 (11)	
H1A	0.6541	0.9443	0.4352	0.057*	
C2	0.7803 (8)	1.1222 (5)	0.4906 (3)	0.0562 (13)	
H2A	0.8212	1.1006	0.5478	0.067*	
C3	0.8193 (7)	1.2488 (5)	0.4736 (3)	0.0482 (11)	
H3A	0.8847	1.3138	0.5188	0.058*	
C4	0.7579 (6)	1.2732 (4)	0.3883 (3)	0.0341 (9)	
C5	0.7763 (6)	1.3928 (4)	0.3402 (3)	0.0389 (10)	
C6	0.6842 (6)	1.3482 (4)	0.2420 (3)	0.0354 (9)	
C7	0.6627 (6)	1.4071 (4)	0.1647 (3)	0.0451 (11)	
H7A	0.7073	1.4946	0.1656	0.054*	
C8	0.5715 (7)	1.3310 (5)	0.0844 (3)	0.0487 (11)	
H8	0.5559	1.3670	0.0296	0.058*	
C9	0.5033 (6)	1.2005 (4)	0.0861 (3)	0.0404 (10)	
H9A	0.4419	1.1517	0.0316	0.049*	
C10	0.6136 (5)	1.2180 (3)	0.2354 (3)	0.0291 (8)	
C11	0.6594 (6)	1.1723 (4)	0.3241 (3)	0.0320 (8)	
C12	0.3421 (6)	0.6768 (4)	0.2441 (3)	0.0362 (9)	
C13	0.2920 (5)	0.5422 (4)	0.2679 (3)	0.0318 (8)	
C14	0.3450 (6)	0.5166 (4)	0.3568 (3)	0.0362 (9)	
H14A	0.4113	0.5820	0.4014	0.043*	
C15	0.2974 (6)	0.3917 (4)	0.3781 (3)	0.0411 (10)	
H15A	0.3312	0.3737	0.4375	0.049*	
C16	0.2012 (6)	0.2952 (4)	0.3122 (3)	0.0338 (9)	
H16A	0.1704	0.2121	0.3273	0.041*	
C17	0.1493 (5)	0.3197 (3)	0.2233 (3)	0.0276 (8)	
C18	0.1937 (5)	0.4439 (3)	0.2020 (3)	0.0309 (8)	
H19A	0.1573	0.4615	0.1428	0.037*	
C19	0.0452 (5)	0.2137 (3)	0.1493 (3)	0.0311 (8)	

### Atomic displacement parameters (Å^2^)

**Table:**

	*U*^11^	*U*^22^	*U*^33^	*U*^12^	*U*^13^	*U*^23^
Cd1	0.03412 (18)	0.02318 (16)	0.03710 (18)	0.00121 (11)	−0.00345 (12)	0.00067 (11)
O1	0.0498 (19)	0.0243 (15)	0.069 (2)	−0.0032 (13)	−0.0120 (16)	−0.0015 (14)
O2	0.055 (2)	0.0298 (16)	0.063 (2)	0.0004 (14)	−0.0124 (16)	0.0103 (14)
O3	0.0568 (19)	0.0375 (16)	0.0360 (17)	−0.0061 (14)	−0.0068 (14)	−0.0016 (13)
O4	0.0468 (18)	0.0216 (14)	0.061 (2)	0.0021 (12)	−0.0168 (15)	0.0021 (13)
O5	0.0478 (18)	0.0318 (15)	0.0423 (17)	0.0043 (13)	−0.0111 (13)	−0.0030 (12)
O6	0.0420 (16)	0.0411 (16)	0.0401 (16)	0.0089 (13)	0.0007 (13)	0.0081 (13)
O7	0.0371 (16)	0.0300 (15)	0.0556 (18)	0.0009 (12)	0.0049 (14)	−0.0019 (13)
O8	0.0515 (19)	0.0296 (17)	0.076 (2)	0.0010 (14)	−0.0073 (17)	−0.0039 (15)
OW2	0.0510 (19)	0.0481 (19)	0.061 (2)	0.0056 (15)	0.0053 (16)	0.0144 (16)
N1	0.0396 (19)	0.0273 (17)	0.0382 (19)	−0.0008 (14)	0.0019 (15)	0.0008 (14)
N2	0.0299 (17)	0.0321 (18)	0.0330 (18)	0.0024 (14)	−0.0027 (14)	−0.0014 (14)
C1	0.067 (3)	0.038 (2)	0.039 (2)	0.009 (2)	0.002 (2)	0.011 (2)
C2	0.069 (3)	0.060 (3)	0.036 (3)	0.009 (3)	−0.006 (2)	0.010 (2)
C3	0.049 (3)	0.051 (3)	0.038 (2)	0.002 (2)	−0.005 (2)	−0.007 (2)
C4	0.027 (2)	0.034 (2)	0.036 (2)	0.0024 (16)	0.0011 (16)	−0.0111 (17)
C5	0.028 (2)	0.029 (2)	0.055 (3)	0.0015 (17)	0.0003 (18)	−0.0055 (19)
C6	0.027 (2)	0.0250 (19)	0.053 (3)	0.0035 (16)	0.0016 (18)	0.0025 (18)
C7	0.039 (2)	0.031 (2)	0.065 (3)	0.0043 (19)	0.002 (2)	0.013 (2)
C8	0.040 (2)	0.062 (3)	0.050 (3)	0.018 (2)	0.004 (2)	0.020 (2)
C9	0.039 (2)	0.044 (2)	0.036 (2)	0.0053 (19)	0.0006 (18)	0.0020 (19)
C10	0.0218 (18)	0.0279 (19)	0.036 (2)	0.0036 (15)	0.0032 (15)	−0.0014 (16)
C11	0.030 (2)	0.027 (2)	0.036 (2)	0.0012 (16)	0.0006 (16)	−0.0024 (16)
C12	0.030 (2)	0.025 (2)	0.051 (3)	0.0025 (17)	0.0037 (19)	0.0002 (18)
C13	0.0259 (19)	0.027 (2)	0.041 (2)	0.0049 (16)	0.0000 (16)	0.0001 (16)
C14	0.039 (2)	0.030 (2)	0.037 (2)	0.0061 (17)	−0.0036 (18)	−0.0050 (17)
C15	0.045 (2)	0.045 (2)	0.034 (2)	0.011 (2)	−0.0019 (18)	0.0073 (19)
C16	0.035 (2)	0.0262 (19)	0.042 (2)	0.0084 (17)	0.0033 (17)	0.0063 (17)
C17	0.0238 (18)	0.0235 (18)	0.035 (2)	0.0036 (15)	0.0008 (15)	0.0021 (15)
C18	0.028 (2)	0.0264 (19)	0.036 (2)	0.0041 (16)	0.0003 (16)	0.0010 (16)
C19	0.0247 (19)	0.024 (2)	0.043 (2)	0.0039 (15)	0.0011 (17)	−0.0023 (17)

### Geometric parameters (Å, °)

**Table:**

Cd1—O7	2.271 (3)	C1—H1A	0.9300
Cd1—O6	2.326 (3)	C2—C3	1.388 (7)
Cd1—O2	2.354 (3)	C2—H2A	0.9300
Cd1—O5	2.368 (3)	C3—C4	1.356 (6)
Cd1—O1	2.441 (3)	C3—H3A	0.9300
Cd1—N2	2.472 (3)	C4—C11	1.388 (5)
Cd1—N1	2.492 (3)	C4—C5	1.517 (6)
Cd1—C12	2.736 (4)	C5—C6	1.514 (6)
O1—C12	1.254 (5)	C6—C7	1.361 (6)
O2—C12	1.246 (5)	C6—C10	1.381 (5)
O3—C19	1.238 (5)	C7—C8	1.388 (7)
O4—C19	1.262 (5)	C7—H7A	0.9300
O5—HO5A	0.8494	C8—C9	1.394 (6)
O5—HO5B	0.9593	C8—H8	0.9300
O6—HO6A	0.9221	C9—H9A	0.9300
O6—HO6B	0.9864	C10—C11	1.466 (6)
O7—HO7A	0.9108	C12—C13	1.505 (5)
O7—HO7B	0.9138	C13—C18	1.384 (5)
O8—C5	1.203 (5)	C13—C14	1.390 (6)
OW1—HW1A	0.8890	C14—C15	1.393 (6)
OW1—HW1B	0.8979	C14—H14A	0.9300
OW2—HW2A	0.9870	C15—C16	1.369 (6)
OW2—HW2B	0.9517	C15—H15A	0.9300
N1—C11	1.324 (5)	C16—C17	1.385 (5)
N1—C1	1.333 (5)	C16—H16A	0.9300
N2—C10	1.321 (5)	C17—C18	1.385 (5)
N2—C9	1.332 (5)	C17—C19	1.514 (5)
C1—C2	1.388 (7)	C18—H19A	0.9300
			
O7—Cd1—O6	174.04 (10)	C4—C3—H3A	121.5
O7—Cd1—O2	94.34 (11)	C2—C3—H3A	121.5
O6—Cd1—O2	89.02 (11)	C3—C4—C11	118.9 (4)
O7—Cd1—O5	99.74 (11)	C3—C4—C5	134.3 (4)
O6—Cd1—O5	85.55 (10)	C11—C4—C5	106.8 (4)
O2—Cd1—O5	82.74 (10)	O8—C5—C6	127.8 (4)
O7—Cd1—O1	88.67 (11)	O8—C5—C4	126.7 (4)
O6—Cd1—O1	89.30 (11)	C6—C5—C4	105.5 (3)
O2—Cd1—O1	54.28 (10)	C7—C6—C10	118.1 (4)
O5—Cd1—O1	136.81 (10)	C7—C6—C5	134.3 (4)
O7—Cd1—N2	83.60 (10)	C10—C6—C5	107.6 (3)
O6—Cd1—N2	95.28 (10)	C6—C7—C8	117.1 (4)
O2—Cd1—N2	156.30 (11)	C6—C7—H7A	121.4
O5—Cd1—N2	74.40 (10)	C8—C7—H7A	121.4
O1—Cd1—N2	148.78 (10)	C7—C8—C9	119.9 (4)
O7—Cd1—N1	90.91 (11)	C7—C8—H8	120.1
O6—Cd1—N1	83.18 (11)	C9—C8—H8	120.1
O2—Cd1—N1	131.53 (11)	N2—C9—C8	123.6 (4)
O5—Cd1—N1	143.43 (10)	N2—C9—H9A	118.2
O1—Cd1—N1	77.78 (10)	C8—C9—H9A	118.2
N2—Cd1—N1	72.17 (11)	N2—C10—C6	127.1 (4)
O7—Cd1—C12	92.22 (11)	N2—C10—C11	123.2 (3)
O6—Cd1—C12	88.55 (11)	C6—C10—C11	109.7 (3)
O2—Cd1—C12	27.01 (11)	N1—C11—C4	126.0 (4)
O5—Cd1—C12	109.62 (11)	N1—C11—C10	123.7 (3)
O1—Cd1—C12	27.27 (11)	C4—C11—C10	110.3 (3)
N2—Cd1—C12	174.70 (11)	O2—C12—O1	122.2 (4)
N1—Cd1—C12	104.75 (12)	O2—C12—C13	119.4 (4)
C12—O1—Cd1	89.6 (3)	O1—C12—C13	118.4 (4)
C12—O2—Cd1	93.9 (2)	O2—C12—Cd1	59.1 (2)
Cd1—O5—HO5A	115.3	O1—C12—Cd1	63.1 (2)
Cd1—O5—HO5B	111.3	C13—C12—Cd1	177.9 (3)
HO5A—O5—HO5B	110.7	C18—C13—C14	119.9 (4)
Cd1—O6—HO6A	117.7	C18—C13—C12	120.4 (4)
Cd1—O6—HO6B	104.4	C14—C13—C12	119.6 (4)
HO6A—O6—HO6B	109.4	C13—C14—C15	119.1 (4)
Cd1—O7—HO7A	122.1	C13—C14—H14A	120.4
Cd1—O7—HO7B	120.4	C15—C14—H14A	120.4
HO7A—O7—HO7B	117.4	C16—C15—C14	120.5 (4)
HW1A—OW1—HW1B	121.2	C16—C15—H15A	119.8
HW2A—OW2—HW2B	93.1	C14—C15—H15A	119.8
C11—N1—C1	114.5 (4)	C15—C16—C17	120.7 (4)
C11—N1—Cd1	109.7 (3)	C15—C16—H16A	119.6
C1—N1—Cd1	135.7 (3)	C17—C16—H16A	119.6
C10—N2—C9	114.2 (3)	C16—C17—C18	119.1 (3)
C10—N2—Cd1	110.7 (2)	C16—C17—C19	121.4 (3)
C9—N2—Cd1	135.1 (3)	C18—C17—C19	119.5 (3)
N1—C1—C2	124.0 (4)	C13—C18—C17	120.6 (4)
N1—C1—H1A	118.0	C13—C18—H19A	119.7
C2—C1—H1A	118.0	C17—C18—H19A	119.7
C1—C2—C3	119.7 (4)	O3—C19—O4	123.9 (4)
C1—C2—H2A	120.2	O3—C19—C17	118.5 (3)
C3—C2—H2A	120.2	O4—C19—C17	117.5 (3)
C4—C3—C2	117.0 (4)		

### Hydrogen-bond geometry (Å, °)

**Table:**

*D*—H···*A*	*D*—H	H···*A*	*D*···*A*	*D*—H···*A*
O5—HO5A···O3^i^	0.85	1.95	2.728 (4)	151
O5—HO5B···O4^ii^	0.96	2.06	2.933 (4)	151
O6—HO6A···OW1^iii^	0.92	1.86	2.776 (5)	175
O6—HO6B···O4^ii^	0.99	1.70	2.675 (4)	171
O7—HO7A···O4^iv^	0.91	1.96	2.744 (4)	143
O7—HO7B···OW2	0.91	1.87	2.757 (4)	162
OW1—HW1A···O8^v^	0.89	2.07	2.903 (5)	156
OW1—HW1B···O1^vi^	0.90	2.12	2.824 (5)	135
OW2—HW2A···O3^vii^	0.99	1.80	2.769 (4)	168
OW2—HW2B···O2^viii^	0.95	1.99	2.936 (5)	173

Symmetry codes: (i) −*x*, −*y*+1, −*z*; (ii) *x*, *y*+1, *z*; (iii) −*x*, −*y*+1, −*z*+1; (iv) *x*+1, *y*+1, *z*; (v) −*x*+1, −*y*+2, −*z*+1; (vi) −*x*+1, −*y*+1, −*z*+1; (vii) −*x*+1, −*y*+1, −*z*; (viii) *x*+1, *y*, *z*.
